# Adverse perception of cough in patients with severe asthma: a discrete choice experiment

**DOI:** 10.1183/23120541.00442-2022

**Published:** 2023-01-03

**Authors:** Joshua Holmes, Vikki O'Neill, Lorcan P. McGarvey, Liam G. Heaney

**Affiliations:** 1 Wellcome-Wolfson Institute for Experimental Medicine, Queen's University Belfast, Belfast, UK; 2 Centre for Medical Education, Queen's University Belfast, Belfast, UK

## Abstract

**Background:**

Asthma symptoms adversely impact quality of life in particular in those with poor disease control. Commonly used patient-reported measures for asthma used to assess asthma control often inadequately capture the impact of cough, despite evidence that cough is one of the most bothersome symptoms for patients with asthma. This study aims to improve our understanding of how patients with asthma perceive cough to better understand its clinical impact.

**Methods:**

A discrete choice experiment (DCE) was performed in two distinct adult asthma populations; those with severe asthma as defined by Global Initiative for Asthma (GINA) step 4/5 classification and those with moderate asthma (a GINA steps 2 or 3 classification of asthma severity).

**Results:**

Choices were highly dominated by the cough attribute in the symptoms complexes; 48.4% of patients with severe asthma and 31.3% with moderate asthma consistently chose the alternative with the lowest level of cough. Furthermore, cough predominance was found to be significantly associated with severity of asthma (p=0.047). Patients with moderate asthma were not willing to accept any additional symptoms to reduce cough from severe to mild. However, these patients were willing to accept mild breathlessness, mild sleep disturbance, severe chest tightness and severe wheezing to remove coughing altogether.

**Conclusions:**

Patients with asthma prefer to have less cough and are willing to accept greater levels of other symptoms to achieve this. Additionally, asthma severity may influence an individual's perception of their symptoms; cough is a more important symptom for patients with severe asthma than those with a milder disease.

## Abstract 2

**This study identifies that cough is one of the most bothersome symptoms for asthmatic patients. Compared to other asthma symptoms, patients are willing to trade off less cough with increasing levels of these other symptoms.** https://bit.ly/3Td2wC1

## Introduction

Asthma is a respiratory condition that affects ∼300 million people worldwide and is characterised by a spectrum of variable symptoms including shortness of breath, chest tightness, wheeze and cough and which is usually associated with variable airflow limitation [1]. Often triggered by a range of external stimuli, symptoms tend to vary in frequency and intensity over time [1]. Asthma symptoms impact adversely on quality of life, in particular in those with poor disease control [2], making it a vital consideration when managing the disease. Asthma is also associated with acute deteriorations in condition with increasing symptoms, often referred to as “exacerbations”, which can be very serious events. In addition to negatively affecting a patient's quality of life, these attacks are also associated with increased healthcare utilisation meaning they have a significant economic impact [3].

Current treatment strategies for asthma follow a step-wise escalation of inhaled anti-inflammatory and bronchodilator therapies in response to uncontrolled symptoms and can include add-on treatments, such as maintenance oral corticosteroids or biologic therapies for those with more severe disease. Despite this approach, a number of asthmatic patients remain symptomatic even after being treated with high-dose therapy and are deemed as having “difficult to control” asthma [1].

Asthma symptom control is variable over time and is often monitored using validated patient-reported outcome (PRO) tools. These measures are useful for assessing individual asthma control and for monitoring a patient's progress as treatment is adjusted to manage their asthma. The impact of cough may not be routinely considered by clinicians and is inadequately captured in current measures of disease control. For example, the Asthma Control Questionnaire (ACQ) developed by Juniper *et al.* [4] is one of the most widely used asthma assessment tools but does not consider cough. Similarly, the Asthma Quality of Life Questionnaire has only one of its 32 items relating to cough [5] and the Asthma Control Tool discusses cough within a broad range of asthma symptoms (wheezing, coughing, shortness of breath, chest tightness or pain) over a 4-week period [6]. This is at odds with evidence which suggests that cough is one of the most bothersome symptoms for asthmatic patients [7, 8] and can be indicative of poor asthma control [9]. Osman *et al*. [7] used a postal survey and conjoint analysis to investigate how patients with asthma attending a hospital clinic weighted the importance of different asthma symptoms. They considered the symptoms cough, wheeze, chest tightness, breathlessness and sleep disturbance. Within the study, cough and breathlessness were each found to be twice as important to patients as other symptoms.

Therefore, the aim of this study was to use common asthma symptoms as part of a discrete choice experiment (DCE) [10]. DCEs are commonly used to assess an individual's preferences or choices and allow researchers to gain a more comprehensive insight into the behavioural responses of the study participants enabling a better understanding of how patients view their symptoms [11]. Within a DCE, participants are presented with a series of hypothetical scenarios. In each scenario, the participant is asked to choose their most preferred option from the different alternatives shown. Each of the alternatives comprise different levels of specific attributes. When making their choice between the competing alternatives, participants are “trading off” the attributes, consequently revealing their preferences for these attributes and their levels [12]. Within this study, we assessed responses from two distinct asthma populations; those with severe asthma as defined by Global Initiative for Asthma (GINA) step 4/5 classification and those with moderate asthma (as defined by GINA steps 2 or 3 classification of asthma severity).

## Methods

### Study population

Adult patients, aged 18–75 years, were recruited from two distinct asthma populations. Patients with severe asthma were recruited through a regional tertiary care severe asthma service (Belfast City Hospital) and had severe asthma as defined by GINA step 4/5 classification of asthma severity (step 4: medium-dose inhaled corticosteroid (ICS)/long-acting bronchodilator inhaler (LABA), referred for expert advice; step 5: referred for phenotypic assessment ± add-on treatment [1]). Patients with moderate asthma (GINA steps 2 or 3) (step 2: daily low-dose ICS; step 3: low-dose ICS/LABA or medium-dose ICS) were recruited from primary care. Participants in both cohorts were sent letters with patient information sheets from their clinical team *via* post in advance of a routine clinical assessment and provided written fully informed consent to take part in the study. Patients were consecutively approached to participate in this study

A “rule of thumb” sample size calculation for discrete choice experiments as proposed by Johnson and Orme [13] was used as described below.

The sample size for the main effects depends on the number of choice tasks (*t*), the number of alternatives (*a*) and maximum number of levels of any of the attributes (*c*) according to the following equation:

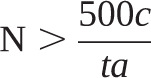
For the purpose of this study *t*=8, *a*=3 and *c*=3:

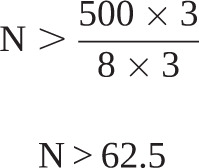
N > 62.5

Therefore, we proposed a plan to recruit at least 60 and up to 100 patients from each group (200 in total) in order to satisfy this sample size requirement

The study was approved by the London – Hampstead Research Ethics Committee (REC reference 19/LO0171).

### Outcome variables

Patients were invited to complete a questionnaire during their study visit. The questionnaire was initially explained to the patient, who was then given time to complete it independently. Demographic information on respondent characteristics was collected, including age, sex, height, weight and current asthma medications.

**FIGURE 1:**
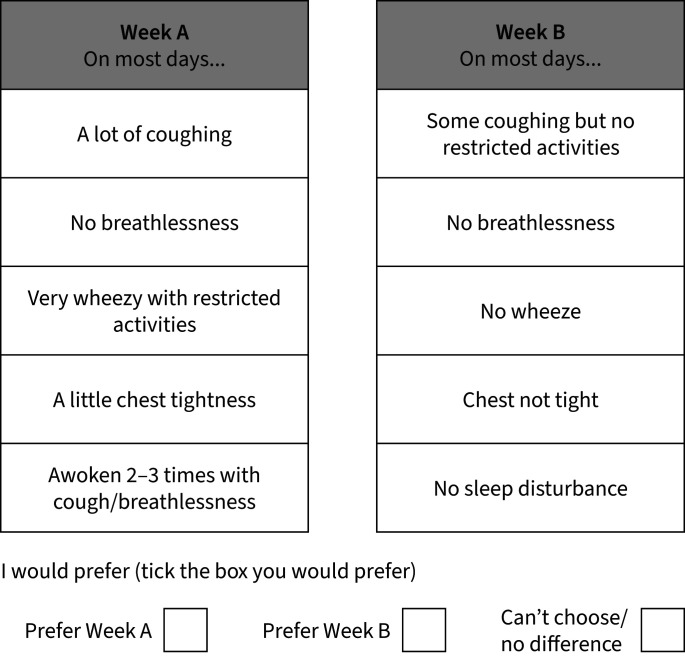
Example layout of a discrete choice scenario.

In addition to the demographic details, the questionnaire also included an Asthma Control Questionnaire (ACQ-5) and DCE. The DCE part of the questionnaire consisted of eight scenarios, each containing two alternatives, “Week A” and “Week B”. The alternatives comprised five different asthma symptoms (attributes), where the specified symptom level represented what that asthma symptom would be like during the week. Table 1 contains the list of asthma symptoms described in each scenario and their corresponding attribute levels. Patients were asked to review each scenario and to choose whether they would prefer to have the symptoms described in either “Week A” or “Week B”. A third option of “can't choose/no difference” was also available (figure 1).

**TABLE 1:** Attribute levels used in the discrete choice experiment

**Symptom**	**Attribute levels**
**Cough**	(0) No cough
	(1) Some coughing but no restricted activities
	(2) A lot of coughing with restricted activities
**Breathlessness**	(0) No breathlessness
	(1) A little breathlessness but no restricted activities
	(2) Very breathless with restricted activities
**Wheeze**	(0) No wheeze
	(1) Some wheezing but with no restricted activities
	(2) Very wheezy with restricted activities
**Chest tightness**	(0) Chest not tight
	(1) A little tightness
	(2) Chest very tight
**Sleep disturbance**	(0) No sleep disturbance
	(1) Awoke once with cough/breathlessness
	(2) Awoken 2–3 times with cough/breathlessness

### Discrete choice experiment

Typically, DCEs are used in health economics to assess and value outcome measures that can be described by a set of attributes or characteristics, which can be further categorised into levels. Participants are given a number of hypothetical scenarios, with each having at least two alternative choices between which a participant must choose their preferred option. For example, this could be a particular health service or treatment procedure that can be described by a set of attributes (length of treatment, cost, waiting time and outcomes). Alternatives are distinguished by their differing attribute levels, often described as a “low”, “medium” and “high”. From the responses to the scenarios, it is then possible to determine which attributes are most important to a participant and to what extent they are willing to “trade off” other attributes in order to achieve a higher level of their most favourable attribute.

### Statistical analysis

#### Demographic data

Demographic differences between the patient groups were tested for using SPSS (SPSS Inc., Chicago, IL, USA). A 5% significance level was used. Histograms and normality plots were used to assess continuous data for normality prior to analysis. Similarly, measures of skew and kurtosis were assessed using Kolmogorov–Smirnov tests. Chi-squared tests were used for categorical data and t-tests/Mann-Whitney U-tests were used, as appropriate, for continuous data.

#### DCE data

For the DCE, a multinomial logit model (MNL) [11] was used to evaluate the strength of respondent preferences for each attribute [11]. All of the attributes listed in table 1, namely, cough, breathlessness, wheeze, chest tightness and sleep disturbance, were included in the models. Data were analysed using Biogeme software (Ecole Polytechnique Fédérale de Lausanne, Switzerland). Raw questionnaire responses were also assessed for lexicographic preferences [14], determining the proportion of patients who always preferred an alternative with the lowest level of a specific symptom, regardless of the other symptoms. The willingness of patients to trade off having greater levels of other symptoms in favour of reducing cough was assessed using marginal rates of substitution. Further details on the DCE methodology and MNL models used are listed in the supplementary material.

## Results

### Patient demographics

A total of 128 asthma patients (64 severe asthma and 64 moderate asthma) were recruited to this study. Patient groups were well matched in terms of age and body mass index (table 2). Patients with severe asthma were more likely to be female (76.6% *versus* 57.8%, p=0.02), more symptomatic (ACQ-5 score 2.3 *versus* 1.6, p=0.04) and receive higher doses of inhaled corticosteroids (BDP equivalent 2000 μg *versus* 800 μg, p<0.001) when compared to patients with moderate asthma. For the severe asthma patients, 61 exhaled nitric oxide fraction (*F*_ENO_) measurements and 32 blood eosinophil counts were also available.

**TABLE 2:** Respondent demographics

**Demographic**	**Severe asthma**	**Moderate asthma**
**Subjects n**	64	64
**Age years**	56.0 (46.0–61.0)	56.0 (46.3–62.0)
**Female**	49 (76.6)	37 (57.8)
**BMI kg·m^−2^**	29.3 (26.4–34.7)	28.2 (25.3–32.7)
**ACQ-5 score**	2.3 (1.1–3.6)	1.6 (0.8–3.0)
**BDP equivalent dose µg**	2000 (2000–2000)	800 (400–1000)
**FEV_1_ % predicted**	86.6 (74.8–96.4)	-
***F*_ENO_ ppb**	26.0 (13.5–39.5)	-
**Blood eosinophil count (cells·µL^−1^)**	260 (120–450)	-

Data presented as n, median (IQR) or n (%). BMI: body mass index; ACQ-5: Asthma Control Questionnaire 5; FEV_1_: forced expiratory volume in 1 s; *F*_ENO_: exhaled nitric oxide fraction.

### MNL models for severe and mild asthma

The results of the MNL models are presented in tables 3 (severe asthma patients) and 4 (moderate asthma patients). In patients with severe asthma, for all symptoms, there was a significant difference between patient's preferences for level 0 and level 2, where patients preferred level 0. For breathlessness, a significant difference was also found between preferences for level 0 (No breathlessness) and level 1 (A little breathlessness but no restricted activitie*s*). For sleep disturbance, the difference in preferences between levels 0 and 1 was not found to be significant at the 5% level. The coefficient for “No preference between scenarios” (DK) was negative and highly significant, meaning that patients preferred to make a choice between the two DCE scenarios rather than be indecisive. In all demographic comparisons, except ICS dose (Model 6), patient preferences for the cough attribute levels were not found to be significantly different.

**TABLE 3:** Results for multinomial logit (MNL) models in patients with severe asthma

	**Model 1: MNL**		**Model 2: Sex**		**Model 3: ACQ_1.5_**		**Model 4: Age_50_**	
**LL(0)**	−562.49		−562.49		−562.49		−562.49	
**LL(β)**	−273.58		−273.38		−273.17		−273.57	
**Parameters**	9		11		11		11	
**Adj. *p^2^***	0.498		0.494		0.495		0.494	
***n***	64		64		64		64	
	**Est.**	**|t-rat|**	**Est.**	**|t-rat|**	**Est.**	**|t-rat|**	**Est.**	**|t-rat|**
**β Breath_L1_**	**−0.84**	3.04	**−0.85**	3.05	**−0.84**	3.05	**−0.84**	3.04
**β Breath_L2_**	**−1.98**	8.36	**−1.98**	8.34	**−1.98**	8.39	**−1.98**	8.38
**β Sleep_L1_**	−0.37	1.74	−0.38	1.73	−0.37	1.74	−0.37	1.74
**β Sleep_L2_**	**−1.02**	2.35	**−1.03**	2.35	**−1.02**	2.36	**−1.02**	2.36
**β Tight_L2_**	**−1.57**	4.41	**−1.57**	4.40	**−1.57**	4.46	**−1.57**	4.42
**β Wheeze_L2­_**	**−1.17**	4.68	**−1.17**	4.69	**−1.18**	4.71	**−1.17**	4.69
**β Cough_L1_**	**−0.63**	2.31	**-**	**-**	**-**	**-**	**-**	**-**
**β Cough_L2_**	**−1.86**	7.16	**-**	**-**	**-**	**-**	**-**	**-**
**β DK**	**−4.47**	9.06	**−4.48**	9.01	**−4.48**	9.12	**−4.47**	9.08
**β Cough_L1 Baseline_**	**-**	**-**	−0.73	1.54	**-**	**-**	**-**	**-**
**Δ Cough_L1 Female_**	**-**	**-**	0.13	0.25	**-**	**-**	**-**	**-**
**β Cough_L2 Baseline_**	**-**	**-**	**−1.73**	5.31	**-**	**-**	**-**	**-**
**Δ Cough_L2 Female_**	**-**	**-**	−0.18	0.44	**-**	**-**	**-**	**-**
**β Cough_L1 Baseline_**	**-**	**-**	**-**	**-**	−0.65	1.30	**-**	**-**
**Δ Cough_L1 ACQ >1.5_**	**-**	**-**	**-**	**-**	0.03	0.05	**-**	**-**
**β Cough_L2 Baseline_**	**-**	**-**	**-**	**-**	**−2.07**	5.02	**-**	**-**
**Δ Cough_L2 ACQ >1.5_**	**-**	**-**	**-**	**-**	0.30	0.66	**-**	**-**
**β Cough_L1 Baseline_**	**-**	**-**	**-**	**-**	**-**	**-**	−0.60	1.24
**Δ Cough_L1 Age >50_**	**-**	**-**	**-**	**-**	**-**	**-**	−0.06	0.12
**β Cough_L2 Baseline_**	**-**	**-**	**-**	**-**	**-**	**-**	**−1.85**	4.54
**Δ Cough_L2 Age > 50_**	**-**	**-**	**-**	**-**	**-**	**-**	−0.01	0.03

	**Model 5: BMI_30_**		**Model 6: BDP_1000_**		**Model 7: FENO_20_**		**Model 8: Blood Eos_150_**	
**LL(0)**	−553.70		−553.70		−536.12		−281.25	
**LL(β)**	−269.88		−268.13		−262.77		−140.80	
**Parameters**	11		11		11		11	
**Adj. *p^2^***	0.493		0.496		0.489		0.460	
***n***	63		63		61		32	
	**Est.**	**|t-rat|**	**Est.**	**|t-rat|**	**Est.**	**|t-rat|**	**Est.**	**|t-rat|**
**β Breath_L1_**	**−0.80**	2.89	**−0.85**	3.02	**−0.77**	2.68	**−1.02**	2.76
**β Breath_L2_**	**−1.97**	8.21	**−2.01**	8.10	**−1.98**	8.26	**−1.96**	6.29
**β Sleep_L1_**	−0.38	1.73	−0.33	1.53	−0.33	1.50	−0.25	0.85
**β Sleep_L2_**	**−1.04**	2.34	**−1.03**	2.28	**−1.02**	2.30	**−**0.42	0.86
**β Tight_L2_**	**−1.55**	4.35	**−1.64**	4.44	**−1.51**	4.22	**−1.57**	3.53
**β Wheeze_L2­_**	**−1.18**	4.65	**−1.18**	4.65	**−1.10**	4.48	**−1.16**	2.94
**β Cough_L1_**	**-**	**-**	**-**	**-**	**-**	**-**	**-**	**-**
**β Cough_L2_**	**-**	**-**	**-**	**-**	**-**	**-**	**-**	**-**
**β DK**	**−4.46**	8.92	**−4.46**	8.86	**−4.36**	8.88	**−4.34**	6.19
**β Cough_L1 Baseline_**	**−0.90**	2.70	**-**	**-**	**-**	**-**	**-**	**-**
**Δ Cough_L1 BMI >30_**	0.63	1.18	**-**	**-**	**-**	**-**	**-**	**-**
**β Cough_L2 Baseline_**	**−1.99**	7.01	**-**	**-**	**-**	**-**	**-**	**-**
**Δ Cough_L2 BMI >30_**	0.28	0.66	**-**	**-**	**-**	**-**	**-**	**-**
**β Cough_L1 Baseline_**	**-**	**-**	**0.63**	2.72	**-**	**-**	**-**	**-**
**Δ Cough_L1 BDP >1000_**	**-**	**-**	**−1.31**	3.00	**-**	**-**	**-**	**-**
**β Cough_L2 Baseline_**	**-**	**-**	**−2.98**	14.08	**-**	**-**	**-**	**-**
**Δ Cough_L2 BDP >1000_**	**-**	**-**	**1.16**	4.53	**-**	**-**	**-**	**-**
**β Cough_L1 Baseline_**	**-**	**-**	**-**	**-**	−0.51	1.56	**-**	**-**
**Δ Cough_L1_ *F***_**ENO**_ **_≥20_**	**-**	**-**	**-**	**-**	−0.12	0.23	**-**	**-**
**β Cough_L2 Baseline_**	**-**	**-**	**-**	**-**	**−1.91**	5.63	**-**	**-**
**Δ Cough_L2_ * F***_**ENO**_ **_≥20_**	**-**	**-**	**-**	**-**	0.15	0.37	**-**	**-**
**β Cough_L1 Baseline_**	**-**	**-**	**-**	**-**	**-**	**-**	−0.36	0.87
**Δ Cough_L1 Eos ≥150_**	**-**	**-**	**-**	**-**	**-**	**-**	−0.04	0.06
**β Cough_L2 Baseline_**	**-**	**-**	**-**	**-**	**-**	**-**	**−1.56**	3.56
**Δ Cough_L2 Eos ≥150_**	**-**	**-**	**-**	**-**	**-**	**-**	−0.23	0.41

Figures in bold indicate a p-value <0.05; Est.: estimate; |t-rat|: absolute t-ratio; LL(0): null log-likelihood; LL(β): final log-likelihood; Adj. *ρ^2^*: adjusted rho-square; DK: no preference between scenarios; ACQ: Asthma Control Questionnaire; BMI: body mass index; *F*_ENO_: exhaled nitric oxide fraction.

In patients with moderate asthma, for all symptoms, there was a significant difference between patient's preferences for level 0 and level 2, and also level 0 and level 1, where patients always preferred level 0. The coefficient for ‘No preference between scenarios’ (DK) was negative and highly significant. In all demographic comparisons, except sex (Model 2), patient preferences for the cough attribute levels were not found to be significantly different. Males did not have significantly different preferences between level 0 and level 1 cough; however females did distinguish between level 0 and level 1 cough. No differences were found between sex for level 2 cough.

### Attribute dominance

Questionnaire responses were assessed to determine the presence of lexicographic preferences, namely the dominance of a particular attribute (symptom). Scenarios 1 and 3 were excluded from this analysis, as they were designed with an alternative, which was fully dominant; one alternative contained higher attribute levels for all symptoms. Additionally, when assessing the dominance of each attribute, a scenario was excluded if the attribute level was the same in both alternatives. Table 5 shows the proportions of patients who, for each of the attributes, always chose the alternative containing the lowest level of this specific attribute, regardless of the levels of other attributes.

**TABLE 4:** Results for multinomial logit (MNL) models in patients with moderate asthma

	**Model 1: MNL**		**Model 2: Sex**		**Model 3: ACQ_1.5_**	
**LL(0)**	−562.49		−562.49		−562.49	
**LL(β)**	−251.41		−248.18		−250.80	
**Parameters**	9		11		11	
**Adj. *p^2^***	0.537		0.539		0.535	
***n***	64		64		64	
	**Est.**	**|t-rat|**	**Est.**	**|t-rat|**	**Est.**	**|t-rat|**
**β Breath_L1_**	**−1.19**	4.66	**−1.23**	4.83	**−1.20**	4.74
**β Breath_L2_**	**−2.19**	7.91	**−2.26**	8.29	**−2.20**	7.99
**β Sleep_L1_**	**−1.68**	6.32	**−1.73**	6.43	**−1.68**	6.40
**β Sleep_L2_**	**−2.82**	5.99	**−2.93**	6.34	**−2.82**	6.05
**β Tight_L2_**	**−1.64**	4.00	**−1.71**	4.08	**−1.64**	4.02
**β Wheeze_L2­_**	**−1.61**	5.63	**−1.64**	5.66	**−1.61**	5.63
**β Cough_L1_**	**−1.06**	1.96	**-**	**-**	**-**	**-**
**β Cough_L2_**	**−1.90**	7.33	**-**	**-**	**-**	**-**
**β DK**	**−6.45**	10.27	**−6.59**	10.50	**−6.46**	10.25
**β Cough_L1 Baseline_**	**-**	**-**	0.34	0.53	**-**	**-**
**Δ Cough_L1 Female_**	**-**	**-**	**−1.95**	2.34	**-**	**-**
**β Cough_L2 Baseline_**	**-**	**-**	**−1.94**	5.45	**-**	**-**
**Δ Cough_L2 Female_**	**-**	**-**	−0.001	0.53	**-**	**-**
**β Cough_L1 Baseline_**	**-**	**-**	**-**	**-**	−1.10	1.50
**Δ Cough_L1 ACQ >1.5_**	**-**	**-**	**-**	**-**	0.07	0.12
**β Cough_L2 Baseline_**	**-**	**-**	**-**	**-**	**−2.07**	6.78
**Δ Cough_L2 ACQ >1.5_**	**-**	**-**	**-**	**-**	0.32	0.93

	**Model 4** **: Age_50_**		**Model 5: BMI_30_**		**Model 6: BDP_1000_**	
**LL(0)**	−562.49		−562.49		−553.70	
**LL(β)**	−250.43		−245.91		−248.72	
**Parameters**	11		11		11	
**Adj. *p^2^***	0.535		0.530		0.530	
***N***	64		62		63	
	**Est.**	**|t-rat|**	**Est.**	**|t-rat|**	**Est.**	**|t-rat|**
**β Breath_L1_**	**−1.21**	4.55	**−1.22**	4.67	**−1.20**	4.70
**β Breath_L2_**	**−2.21**	7.85	**−2.14**	7.87	**−2.18**	7.90
**β Sleep_L1_**	**−1.69**	6.41	**−1.68**	6.37	**−1.70**	6.42
**β Sleep_L2_**	**−2.84**	5.86	**−2.79**	5.96	**−2.81**	6.06
**β Tight_L2_**	**−1.65**	4.03	**−1.56**	3.86	**−1.62**	3.98
**β Wheeze_L2­_**	**−1.62**	5.57	**−1.55**	5.68	**−1.62**	5.64
**β Cough_L1_**	**-**	**-**	**-**	**-**	**-**	**-**
**β Cough_L2_**	**-**	**-**	**-**	**-**	**-**	**-**
**β DK**	**−6.47**	10.07	**−6.40**	10.27	**−6.42**	10.29
**β Cough_L1 Baseline_**	**−1.51**	2.02	**-**	**-**	**-**	**-**
**Δ Cough_L1 Age >50_**	0.76	1.16	**-**	**-**	**-**	**-**
**β Cough_L2 Baseline_**	**−1.86**	4.94	**-**	**-**	**-**	**-**
**Δ Cough_L2 Age >50_**	−0.08	0.21	**-**	**-**	**-**	**-**
**β Cough_L1 Baseline_**	**-**	**-**	−0.93	1.43	**-**	**-**
**Δ Cough_L1 BMI >30_**	**-**	**-**	−0.31	0.49	**-**	**-**
**β Cough_L2 Baseline_**	**-**	**-**	**−1.79**	5.68	**-**	**-**
**Δ Cough_L2 BMI >30_**	**-**	**-**	−0.23	0.64	**-**	**-**
**β Cough_L1 Baseline_**	**-**	**-**	**-**	**-**	**−1.05**	2.05
**Δ Cough_L1 BDP >1000_**	**-**	**-**	**-**	**-**	0.03	0.05
**β Cough_L2 Baseline_**	**-**	**-**	**-**	**-**	**−1.78**	5.89
**Δ Cough_L2 BDP >1000_**	**-**	**-**	**-**	**-**	−0.19	0.55

Figures in bold indicate a p-value <0.05; Est.: estimate; |t-rat|: absolute t-ratio; LL(0): null log-likelihood; LL(β): final log-likelihood; Adj. *ρ^2^*: adjusted rho-square; DK: no preference between scenarios; MNL: multinomial logit model; ACQ: Asthma Control Questionnaire; BMI: body mass index.

**TABLE 5:** Number (%) of patients whose choices were determined by a dominant attribute

**Attributes**	**Severe asthma^#^**	**Moderate asthma^#^**	**p*-*value**
**Breathlessness**	4 (6.3)	7 (10.9)	0.344
**Sleep disturbance**	1 (1.6)	5 (7.8)	0.094
**Chest tightness**	3 (4.7)	4 (6.3)	0.697
**Wheeze**	0 (0.0)	1 (1.6)	–
**Cough**	31 (48.4)	20 (31.3)	0.047

Differences between patient groups assessed using chi-square test. ^#^: n=64.

**TABLE 6:** Patient willingness to accept symptoms in favour of a reduced cough

	**Severe asthma**		**Moderate asthma**	
	**Cough_L2_ to Cough_L1_**	**Cough_L2_ to Cough_L0_**	**Cough_L2_ to Cough_L1_**	**Cough_L2_ to Cough_L0_**
**Accept Breathlessness_L1_**	1.46	2.21	0.70	1.60
**Accept Breathlessness_L2_**	0.62	0.94	0.38	0.87
**Accept Sleep disturbance_L1_**	3.29	4.97	0.50	1.13
**Accept Sleep disturbance_L2_**	1.21	1.82	0.30	0.67
**Accept Chest tightness_L2_**	0.78	1.18	0.51	1.16
**Accept Wheeze_L2_**	1.05	1.59	0.52	1.18

A value >1 indicates a willingness to move from a current level 0 of the symptom to gain a reduction in cough.

A high number of patients made choices that were dominated by the cough attribute; 48.4% of patients with severe asthma and 31.3% with moderate asthma always choose the alternative with the lowest level of cough. Cough dominance was found to be significantly associated with severity of asthma, where dominance was higher in patients with severe asthma than with moderate asthma (p=0.047). No other symptom dominance was found to be significantly associated with severity of asthma.

### Symptom trade off

The willingness of patients to *accept* a worsening of other symptoms in favour of a reduced cough symptom is shown in table 6. A value >1 indicates a willingness to move from a current level 0 of the symptom to gain a reduction in cough. A reduction in cough was defined as moving from CoughL2 (severe cough) to either CoughL1 (mild cough) or CoughL0 (no cough).

Patients with severe asthma were willing to accept mild breathlessness (level 1) in order to reduce a severe cough; however, they were not prepared to accept severe breathlessness (level 2) for any reduction in cough. Severe asthma patients were willing to accept any level of sleep disturbance or wheeze in order to reduce coughing. However, patients with severe asthma were unwilling to accept severe chest tightness to reduce coughing from severe to mild but were willing to accept it in order to have no coughing.

Patients with moderate asthma were not willing to accept any additional symptoms in order to reduce cough from severe to mild. However, these patients were willing to accept mild breathlessness, mild sleep disturbance, severe chest tightness and severe wheezing to remove coughing altogether. They were not willing to accept severe breathlessness and severe sleep disturbance for no coughing.

## Discussion

The findings from this study support previous findings by Osman *et al*. [7], which highlighted the prominence and troublesome nature of cough compared to other symptoms for patients with asthma. This study extends these findings by exploring this in different severities of asthma and provides evidence that patients with asthma prefer to have less cough and are willing to trade off greater levels of other symptoms to achieve this.

Asthma severity may influence an individual's perception of their symptoms and specifically, cough is a more important symptom for patients with severe asthma than those with a milder disease. The relative importance of cough in patients with severe asthma is consistent with previous findings; however, in the study of Osman *et al.*, both breathlessness and cough were each found to be weighted twice as heavily compared to other symptoms, whereas in this study the dominance of these two symptoms was not as apparent. The Osman study did not report asthma control using a validated PRO and population comparisons are difficult, but differences may reflect that patients in this study with well characterised severe asthma and poor baseline control may have stronger preferences for control across a broader spectrum of asthma symptoms.

While cough was comparatively a more significant symptom in severe asthma compared to moderate asthma, patients with a moderate disease felt more strongly about other asthma symptoms. There were no consistent associations between participant demographics and symptom preferences in either the severe or moderate asthma study groups. In the moderate group, females appeared to have a significantly stronger dislike for level 1 cough compared to males, with no difference observed for level 2 cough. There was no sex difference in the severe asthma group.

We found that for patients with asthma, cough is a dominant factor that influences patient symptom preference. Almost half of severe asthma patients and one third of moderate asthma patients chose scenarios with lower levels of coughing regardless of other symptoms. We noted some differences in perception based on disease severity. Moderate asthma patients were not willing to accept any additional symptoms to move from a severe cough to a milder cough, while those with severe asthma were willing to make some concessions by accepting mild levels of breathlessness and any level of sleep disturbance. In a trade off to remove cough entirely, both severe and moderate patients were prepared to accept mild levels of breathlessness and sleep disturbance and severe levels of wheeze and chest tightness. Taken together these findings highlight the prominence with which patients with asthma perceive cough and consider it a symptom they wish to avoid. The dominance of cough in this study is striking; understanding the relative importance as to how patients with asthma perceive their symptoms, in particular cough, is essential to improve asthma management.

Determining how preferences between patients of different demographic and clinical groups differ is essential for better planning of symptom management. Furthermore, by making use of DCEs our study was able to assess whether patients are willing to accept higher levels of other asthma symptoms in order to reduce the burden of coughing.

A limitation of this study is that blood eosinophil and *F*_ENO_ results were only available for a proportion of patients with severe asthma and for no patients with moderate asthma. Unfortunately, due to the nature of patient recruitment it was not possible to achieve these results at the time of questionnaire completion for all patients. Additionally, the relatively small sample size for this study meant that a limited number of analyses could be run. In future studies, it would be preferred to expand the recruitment to allow for additional investigations such as an analysis according to sex, which would be very useful within the context of cough burden.

In summary, this study found that cough is an important symptom for patients with asthma who, independent of disease severity, are willing to accept additional symptoms to reduce cough severity. These observations reinforce the view that the current approach to assess and record asthma control is limited in scope because of a failure to capture the impact and burden of cough. Developing and validating methodology to address this issue both in the clinical and research setting is a priority.

## Supplementary material

10.1183/23120541.00442-2022.Supp1 **Please note:** supplementary material is not edited by the Editorial Office, and is uploaded as it has been supplied by the author.
Supplementary material 00442-2022.SUPPLEMENT
