# Supplementary material for: Adverse perception of cough in patients with severe asthma: a discrete choice experiment
Source: ERJ Open Res. 2023 Jan 3;9(1):00442-2022. doi: 10.1183/23120541.00442-2022 (PMC9808534; doi:10.1183/23120541.00442-2022)
Supplement: Supplementary file 1 [file 00442-2022.SUPPLEMENT.pdf]

Patient perceptions of symptoms in severe asthma: a discrete choice experiment

Joshua Holmes[1], Vikki O'Neill[2], Lorcan P McGarvey[1], and Liam G Heaney[1]

[1]Wellcome-Wolfson Institute for Experimental Medicine, Queen's University Belfast, Northern Ireland and [2]Centre for Medical Education, Queen's University Belfast, Mulhouse Building, Mulhouse Road, Belfast, BT12, Northern Ireland

SUPPLEMENTARY MATERIAL

Discrete Choice Experiment Methodology and Models

In the MNL specification, the deterministic component of utility (the random component of the utility function follows a type I extreme value distribution) for respondent *n* and alternative *i* in choice task *t* (out of *8*) is written as:

$$V_{int} = \beta_{BreathL1}BreathL1_{int} + \beta_{BreathL2}BreathL2_{int} +$$
$$\beta_{SleepL1}SleepL1_{int} + \beta_{SleepL2}SleepL2_{int} +$$
$$\beta_{TightL1}TightL1_{int} + \beta_{TightL2}TightL2_{int} +$$
$$\beta_{WheezeL1}WheezeL1_{int} + \beta_{WheezeL2}WheezeL2_{int} +$$
$$\beta_{CoughL1}CoughL1_{int} + \beta_{CoughL2}CoughL2_{int} \qquad i = \{1,2\}, \qquad (1)$$
$$V_{3nt} = \beta_{DK}DK_{3nt}, \qquad (2)$$

where, as an example, $CoughL1_{int}$ is set to 1 if alternative *i* contains the Cough level 1 (and is set to 0 if alternative *i* has a Cough level other than 1), and where $\beta_{CoughL1}$ is the associated marginal utility coefficient, which is to be estimated.

Equation 1 shows the utility individual *n* will receive if they select either of the first two alternatives, whereas Equation 2 shows the utility individual *n* will receive through the selection of the '*Don't know'* option (displayed as alternative 3, in this case). The attributes were entered as dummy variables in order to allow us to capture any non-linear preference structure for these attributes, where the 0 level was used as the baseline (i.e. the sensitivity for absence of symptom was fixed to zero). Notably, since the baseline was set to level 0 for each attribute, it would be sensible to expect all of the level 1 and level 2 coefficients to be negative, as it is improbable for a patient to prefer experiencing symptoms to no symptoms. For example, it is unlikely that a patient would prefer Cough Level 2 (*A lot of coughing with restricted activities*) to Cough level 0 (*No coughing*). If a coefficient (e.g., $\beta_{CoughL2}$) is found to be significant, this means that patients' preferences for that level is significantly different to the baseline of level 0.

The specification above assumes that preferences for the different symptom attribute levels are the same for all respondents. As we are interested in whether preferences for cough vary across patients, we can revise our model specification to allow for differences in sensitivities by specific demographics/characteristics. Consider for example, a model, which elicits preference differences between male and female respondents. For each of the cough

levels (other than the baseline 0), we thus estimate a base coefficient, along with offsets for the separate groups (male vs female). This specification is shown in Equation 3, where, for example, $\Delta_{CoughL1;Female}$ shows the shift in the utility for Level 1 Cough for a female respondent relative to a male respondent. The shift parameter represents the difference in preferences between the two groups; where a value of 0 would mean that the two groups have the same preference.

$$
\begin{aligned}
V_{int} = {} & \beta_{BreathL1}BreathL1_{int} + \beta_{BreathL2}BreathL2_{int} + \\
& \beta_{SleepL1}SleepL1_{int} + \beta_{SleepL2}\,SleepL2_{int} + \\
& \beta_{TightL1}\,TightL1_{int} + \beta_{TightL2}\,TightL2_{int} + \\
& \beta_{WheezeL1}\,WheezeL1_{int} + \beta_{WheezeL2}\,WheezeL2_{int} + \\
& \beta_{CoughL1;Baseline}CoughL1_{int} + \Delta_{CoughL1;Female}CoughL1_{int} + \\
& \beta_{CoughL2;Baseline}CoughL2_{int} + \Delta_{CoughL2;Female}CoughL2_{int} \qquad i = \{1,2\}, \qquad (3)
\end{aligned}
$$

The MNL models estimated are described in Table E1 below. In the primary MNL model (*model 1*), all patients are assumed to have the same preferences for each of the attributes. The remaining MNL models allow for differences in preferences for the Cough attribute levels between groups. Models 2 to 6 test for differences in preferences by gender (male vs female), ACQ-5 score (≤ 1.5 vs >1.5), age (≤ 50 vs >50), BMI (≤ 30 vs >30) and BDP equivalent inhaled corticosteroid (ICS) dose (≤ 1000µg vs >1000µg). Additional models 7 & 8 test for differences based on fractional exhaled nitric oxide (FeNO) (< 20ppb vs ≥20ppb) and blood eosinophil count (< 0.15 x10^9/L vs ≥150 cell/µl) in the severe asthma patient group only.

Table E1: List of MNL models.

Model No.	Name	Description
**1**	Primary MNL model	Preferences assumed to be the same for all respondents.
**2**	Gender model	Preferences for cough allowed to vary by gender; females compared to males (baseline).
**3**	ACQ-5 model	Preferences for cough allowed to vary by asthma control; ACQ-5 score > 1.5 compared to ACQ-5 score ≤ 1.5 (baseline).
**4**	Age model	Preferences for cough allowed to vary by age; age > 50

		years compared to age ≤ 50 years (baseline).
**5**	Body Mass Index (BMI) model	Preferences for cough allowed to vary by BMI; BMI > 30 compared to BMI ≤ 30.
**6**	Inhaled corticosteroid dose model	Preferences for cough allowed to vary by BDP equivalent dose; BDP equiv. > 1000μg compared to BDP equiv. ≤ 1000μg (baseline).
**7**	Fractional exhaled nitric oxide (FeNO) model	Preferences for cough allowed to vary by FeNO level; FeNO ≥ 20ppb compared to FeNO < 20ppb (baseline).
**8**	Peripheral blood eosinophil model	Preferences for cough allowed to vary by blood eosinophil (Eos) count; Eos ≥ 150 cells/μl compared to Eos < 150 cells/μl (baseline).

Note: Models 7 and 8 were assessed only in patients with severe asthma

Estimation of scale factors

As study respondents were recruited from two distinct asthma populations, it is important to determine whether any differences in preferences found are caused by true preference differences or differences in their associated scale factors[13]. Scale heterogeneity (also referred to as heteroskedasicity[14]) refers to heterogeneity in the variance associated with the random component of utility, ε. Thus, we estimate one set of coefficients, β and an additional scale coefficient for the second primary care population, μ_{PC}. The estimation of a scale model was performed as described by Swait and Louviere (1993)[13]. The test statistic retrieved, λ_A = 38.04, is significant at the 5% significance level; we therefore conclude that the two groups have different preferences, and thus should be modelled separately, rather than performing a grouped analysis with all participants.

Multinominal Logit (MNL) models

For the purposes of quality control and to ensure that patients were engaged when completing the questionnaire, patients who answered any of the choice scenarios irrationally were not included for data analysis (table E2). Given that the two groups (severe asthma vs mild/moderate asthma) needed to be estimated separately (see scale analysis above), over-parameterisation was a methodological concern (i.e., estimating too many parameters). Therefore, as level 1 Chest tightness and level 1 Wheeze were found to be not

significant in any of the preliminary models, for these two attributes level 0 and level 1 were combined.

Table E2: Distribution of the attribute levels for each scenario in the discrete choice experiment

	**Week A**	**Week B**
**Scenario**	*Levels of symptoms (cough, breathlessness, wheeze, chestiness, sleep)*	*Levels of symptoms (cough, breathlessness, wheeze, chestiness, sleep)*
**1**	**(2, 0, 2, 1, 2)**	**(1, 0, 0, 0, 0)**
**2**	(0, 1, 1, 1, 0)	(2, 0, 1, 0, 0)
**3**	**(0, 0, 0, 2, 0)**	**(1, 2, 1, 2, 2)**
**4**	(1, 1, 2, 1, 0)	(2, 1, 0, 2, 1)
**5**	(2, 0, 2, 2, 0)	(0, 2, 2, 0, 1)
**6**	(2, 2, 0, 1, 0)	(2, 0, 1, 1, 1)
**7**	(2, 2, 0, 1, 0)	(0, 0, 0, 1, 2)
**8**	(1, 0, 0, 1, 1)	(2, 1, 0, 0, 2)

The distribution of attribute levels for each of the scenarios are shown in Table 2. For the purposes of quality control and to ensure that patients were engaged when completing the questionnaire, scenarios 1 and 3 were included to assess for rational choice behaviour. Namely, the scenarios were set up so that one alternative was an "obvious" better choice in terms of symptom burden. For example, in scenario 1 (as shown in Table 2), patients should always prefer week B to week A. Patients who answered scenarios 1 and 3 irrationally were not included for data analysis.
